# Crude Saponin from *Platycodon grandiflorum* Attenuates Aβ-Induced Neurotoxicity via Antioxidant, Anti-Inflammatory and Anti-Apoptotic Signaling Pathways

**DOI:** 10.3390/antiox10121968

**Published:** 2021-12-09

**Authors:** Yun-Jeong Ji, Sujin Kim, Jwa-Jin Kim, Gwi Yeong Jang, Minho Moon, Hyung Don Kim

**Affiliations:** 1 Department of Herbal Crop Research, National Institute of Horticultural and Herbal Science, Rural Development Administration, Eumsung 27709, Korea; jyj2842@korea.kr (Y.-J.J.); janggy@korea.kr (G.Y.J.); 2 Department of Biochemistry, College of Medicine, Konyang University, 158, Gwanjeodong-ro, Seo-gu, Daejeon 35365, Korea; aktnfl3371@naver.com; 3 Research Institute for Dementia Science, Konyang University, Daejeon 35365, Korea; 4 Departments of Nephrology, School of Medicine, Chungnam National University, Daejeon 35015, Korea; kjj1021@naver.com; 5 Department of Biochemistry, School of Life Sciences, Chungbuk National University, Cheongju 28644, Korea

**Keywords:** Alzheimer’s disease, antioxidant enzymes, apoptosis, hippocampal neuronal cells, oxidative stress, *Platycodon grandiflorum* saponin

## Abstract

Although *Platycodon grandiflorum* saponins exhibit many beneficial biological effects in various diseases and conditions, how they protect nerve cells against neurodegenerative diseases and Alzheimer’s disease (AD) pathology is unknown. We investigated whether *P. grandiflorum* crude saponin (PGS) protects neurons from neurodegeneration caused by amyloid beta (Aβ)-induced oxidative stress. Hippocampal neuron HT-22 cells were used in the in vitro experiment, and AD mice (5XFAD mice) were used as the in vivo model. Intracellular reactive oxygen species (ROS) was stained with DCF-DA and assessed using fluorescence microscopy. To elucidate the mechanism underlying neuroprotection, intracellular protein levels were assessed by western blotting. In 5XFAD mice, an animal model of AD, nerve damage recovery due to the induction of Aβ toxicity was evaluated by histological analysis. PGS attenuates Aβ-induced neurotoxicity by inhibiting Aβ-induced reactive oxygen species (ROS) production and apoptosis in HT-22 cells. Furthermore, PGS upregulated Nrf2-mediated antioxidant signaling and downregulated NF-κB-mediated inflammatory signaling. Additionally, PGS inhibited apoptosis by regulating the expression of apoptosis-associated proteins. In addition, PGS ameliorated Aβ-mediated pathologies, leading to AD-associated cognitive decline. Conclusions: Taken together, these findings suggest that PGS inhibits Aβ accumulation in the subiculum and cerebral cortex and attenuates Aβ toxicity-induced nerve damage in vitro and in vivo. Therefore, PGS is a resource for developing AD therapeutics.

## 1. Introduction

Alzheimer’s disease (AD) is a cognitive-behavioral disorder caused by degenerative changes in the cerebral cortex and hippocampal cells. The main pathological feature is the formation of senile plaques due to excessive accumulation of amyloid-beta (Aβ) [1]. In particular, the accumulation of Aβ induced by amyloid precursor protein (APP), which is highly neurotoxic, releases neurotoxic factors such as reactive oxygen species (ROS), proinflammatory cytokines, and chemokines, leading to neuronal damage. Oxidative stress damage, along with ROS and malondialdehyde (MDA) overproduction and decreased antioxidant enzyme activity, play an important role in the pathogenesis of AD. Aβ_25–35_ induces ROS overproduction, inhibiting the antioxidant enzyme system, resulting in redox imbalance in cells [2]. The localization of Aβ in the mitochondrial membrane can lead to mitochondrial dysfunction, such as glucose metabolism deficiency, inactivation of key enzymes required for oxidative phosphorylation, and mitochondrial reactive free radical accumulation. [3]. In the Aβ-induced mitochondrial apoptosis pathway, B cell CLL/lymphoma-2 (BCL-2) is a key regulator and, when overexpressed, protects neurons from neurotoxic damage. BCL-2-associated x protein (BAX) initiates the conversion of pro-caspase-9 to caspase-3, promoting cytochrome c release, and consequently, induces apoptosis. In addition, poly (ADP-ribose) polymerase-1 (PARP-1), which is involved in DNA repair, is cleaved by caspase-3, leading to apoptosis and neuron death [4,5]. Excessive Aβ accumulation is mainly attributed to aging-related oxidative stress (OS), and defects in the antioxidant defense mechanisms may increase oxidative stress and accelerate Aβ deposition in AD transgenic mice. Reactive microglia and astrocytes, which surround senile plaques and are critical immune cells in the brain, play a neuroprotective role against oxidative stress and inflammation. Astrocytes and microglia activated by Aβ oligomers induce oxidative stress via the generation of ROS and reactive nitrogen species (RNS), exacerbating neuroinflammation. In the early stages of AD, microglia and astrocytes can effectively eliminate the toxic accumulation of Aβ [6,7].

Efforts have been made to elucidate the relationships between Aβ and neuroinflammation in the pathogenesis of AD. Since inflammatory mechanisms are highly interactive and rarely occur in isolation, the anti-inflammatory effect can alleviate various diseases or their symptoms, derived from AD. Although the mechanism by which Aβ triggers the inflammatory process is complex, there is evidence that the peptide triggers the activation of the transcription factor NF-κB [8]. In response to inflammatory stimuli, IκB kinase (IKK) phosphorylates inhibitors of kappa B (IκB) and free nuclear factor kappa B (NF-κB) is translocated to the nucleus, where it binds to the κB binding site in the target gene promoter region. This induces the transcription of pro-inflammatory mediators, including inducible nitric oxide synthase (iNOS), cyclooxygenase-2 (COX-2), tumor necrosis factor-α (TNF-α) and interleukin-1β (IL-1β). Mitogen-activated protein kinases (MAPKs) are also involved in regulating the production of key inflammatory mediators. Exposure to Aβ stimulation activates c-Jun N-terminal kinase (JNK), extracellular signal-regulated kinase (ERK) and p38 MAPK by phosphorylation at sites that control activation of the NF-κB signaling pathway [9,10]. Consequently, increased Aβ accumulation stimulates the aging of neurons and microglia, promoting neuroinflammation and neurodegeneration, leading to the vicious cycle of AD.

The cholinesterase inhibitors donepezil, galantamine and rivastigmine have been approved by the United States Food and Drug Administration (FDA) for treatment aimed at reducing Aβ levels [11]. However, a complete cure is impossible. Many studies suggest that increased Aβ accumulation in AD pathology is responsible for neurodegeneration. Therefore, reducing the accumulation may be an effective strategy to prevent AD progression. Herbal medicines with various ingredients may be advantageous because they provide multi-target control. Research to date has focused on natural antioxidant products to treat AD, such as *Punica granatum* seed, *Cinnamomum zeylanicum* and Emilia cocaine G [12,13,14].

*Platycodon grandiflorum* (PG) is an herbaceous perennial belonging to the family Campanulaceae and has been used as food and medicine since ancient times. PG is rich in fiber and minerals such as potassium and magnesium. PG has an antibacterial effect on bronchial diseases [15], lowers blood sugar [16], improves cholesterol metabolism [17,18] and exerts an antibacterial effect. Studies on obesity [19], cancer cell proliferation [20] and antioxidant effects [21,22] have been reported. The active ingredients of PG are about 20 saponins, including platycodin D [23,24]. According to Yan et al., the saponin contents of Korean and Chinese *Platycodon grandiflorum* were predominantly platycoside E and platycodin D [25]. Similarly, Ha et al. reported high levels of platycoside E, platycodin D and poly-galacin D in PG [26]. Similar to ginseng saponins, *P. grandiflorum* saponins have anti-inflammatory, antibacterial, antioxidant, tonic and analgesic activities, with the structure of oleanane triterpene as an aglycone [27]. *P*. *grandiflorum* saponins exert neuroprotective, antineuroinflammatory and cognitive-enhancing effects [28]. Therefore, in diseases with various pathological features, such as AD, *P. grandiflorum* crude saponin (PGS), which is multi-targeting and multi-functional, may have therapeutic potential. However, no study has examined the therapeutic effect of PGS on Aβ-induced pathology in intraneuronal cells or AD animal models.

We evaluated the effect of PGS on the accumulation of Aβ and Aβ-induced nerve damage in vitro and in vivo to evaluate its therapeutic potential for AD. The effects of PGS on antioxidant, anti-inflammatory and anti-apoptotic signaling pathways in neurons were also investigated to reveal the mechanisms underlying its neuroprotective activity.

## 2. Materials and Methods

### 2.1. Sample Preparation

The roots of a 3-year-old *P. grandiflorum* (cv. Eutteumbaek) plant harvested from Boeun, Chungcheongbuk-do, Korea, in 2018, were used to prepare samples. They were air-dried at 55 °C for 72 h and ground. Powder (10 kg) was extracted twice in 60 L of 70% ethanol for 10 days. After filtration, the extract was evaporated under vacuum and freeze-dried as *P*. *grandiflorum* extract (PGE). PGS was separated from PGE using a separation and purification system (Accelerated Chromatographic Isolation System, Isolera™, Biotage, Stockholm, Sweden). After filling the 500 mL column with GSH-20 resin, 100 mL of PGE solution diluted with distilled water were injected and washed with 3 L of distilled water and 25% ethanol sequentially. Next, 3 L of 95% ethanol were injected to create a saponin layer. The saponin layer was concentrated using a vacuum condenser and lyophilized as PGS (yield 1.22%). Samples were stored at −80 °C until analysis.

### 2.2. Cell Culture

HT22 Hippocampal-derived neurons were purchased from Merck (Darmstadt, Hesse, Germany). HT22 cells were cultured in Dulbecco’s modified Eagle’s medium (DMEM; Gibco, Grand Island, NY, USA), supplemented with 10% fetal bovine serum (FBS) 100 units/mL penicillin and 100 μg/mL streptomycin at 37 °C in a 5% CO_2_ incubator. The medium was replaced every 2 days for subculture. Cells at passages 5–10 were used in all experiments.

### 2.3. Cell Viability Assay

Cell viability was evaluated by MTS (3-(4,5-dimethylthiazol-2-yl)-5-(3-carboxymethoxyphenyl)-2-(4-sulfophenyl)-2H-tetrazolium [Promega]) assay. HT22 cells were seeded in 96-well plates (1 × 10^4^ cells/well) and treated with PGS (5, 10, and 20 µg/mL) for 48 h. To investigate the effect of PGS on Aβ-induced cytotoxicity, HT22 cells were pretreated for 1 h with PGS (5, 10, and 20 μg/mL) and cytotoxicity was induced by treatment with Aβ (Aβ_25–35_, 10 μM) for 48 h. After treatment with MTS and incubation for 1 h, absorbance at 490 nm was measured using a multi-plate reader (BioTek Instruments, Inc., Winooski, VT, USA).

### 2.4. Measurement of Intracellular ROS Generation in HT22 Cells

Intracellular ROS production was analyzed by a dichloro-dihydro-fluorescein diacetate (DCFH-DA) method. HT22 cells (1 × 10^4^) were inoculated in a black 96-well plate and cultured for 24 h. Next, PGS (5, 10 and 20 μg/mL) was added for 24 h. The cells were treated with serum-free medium (SFM) containing 10 μM Aβ for 4 h and then with SFM containing DCF-DA (DCF-DA; Sigma, St. Louis, MO, USA) for 30 min. The cells were washed with phosphate-buffered saline (PBS), and 100 μL of Dulbecco’s phosphate-buffered saline were added to each well. Thereafter, DCF fluorescence was measured at 485 and 535 nm using a multi-plate reader. Images were obtained using a fluorescence microscope (Carl Zeiss, Jena, Germany) to evaluate intracellular ROS production. The experiment was carried out in triplicate.

### 2.5. Western Blot Analysis

HT22 cells were harvested, reacted at 4 °C for 1 h in RIPA buffer (Cell Signaling, Danvers, MA, USA) and centrifuged at 12,000 rpm for 30 min to separate protein. Protein was quantified using a protein assay kit and mixed with Laemmli sample buffer (Bio-Rad, Hercules, CA, USA). Samples with equal amounts of protein were treated with 10% sodium dodecyl sulfate. After separation by electrophoresis in an (SDS)-polyacrylamide gel, samples were transferred to a polyvinylidene difluoride (PVDF) membrane (Millipore, Darmstadt, Germany). Blocking was carried out in 5% bovine serum albumin (GenDEPOT, Katy, TX, USA) for 30 min. The primary antibody was added overnight at 4 °C, followed by the secondary antibody at room temperature for 1 h. After washing with PBS-T and reacting with enhanced chemiluminescence (ECL) solution (Bio-Rad), a ChemiDoc Imaging System (Bio-Rad) was used to visualize the results.

### 2.6. Analysis of Platycoside E and Platycodin D by HPLC-ELSD

Platycoside E and platycodin D are the major physiologically active and indicator *Platycodon grandiflorum* saponins [17]. They were analyzed as described previously [29]. Analysis-grade platycoside E and platycodin D were purchased from Chengdu Biopurify Phytochemical (Chengdu, Sichuan, China). Samples were dissolved in 40 mL of distilled water and degreased with diethyl ether in a separatory funnel. The separated aqueous layer was extracted three times with water-saturated n-butanol. The n-butanol layer was evaporated at 50 °C. The resulting residue was dissolved in methanol and analyzed. The retention times of platycoside E and platycodin D were confirmed by comparison with the corresponding analytical-grade standards by high-performance liquid chromatography (HPLC) using a Waters Alliance 2695 HPLC system (2424 ELSD, Waters, Milford, MA, USA) and C-18 column (Luna C-18, Phenomenex, 250 × 4.6 mm, 5 μm, Torrance, CA, USA). As the mobile phase, water was used as solvent A and acetonitrile as solvent B. The conditions were 21–21% solvent B for 0–3 min, 21–23% solvent B for 3–23 min, 23–24% solvent B for 23–38 min and 24–100% Solvent B for 38–70 min. The analysis was performed using a solvent gradient of 100–100% solvent B for 70–75 min at a flow rate of 1.0 mL/min, using a sample injection volume of 30 μL. The column temperature was maintained at 40 °C. The ELSD conditions were analyzed by maintaining the atomizer temperature at 42 °C, the drift tube temperature at 85 °C, and the N_2_ gas pressure at 50 psi.

### 2.7. Animals and PGS Administration

The 5XFAD mouse (Tg6799; Jackson Laboratory, Bar Harbor, ME, USA) has five mutations related to early onset familial AD. These are in the human PSEN1 gene (M146L and L286V) and human APP gene (SwedishK607N and M671L, FloridaI716V, and LondonV717I). Wild-type (WT) mice were obtained by crossing female B6SJL/F1 and male 5XFAD mice. Mice were classified as WT and 5XFAD by genotyping of tail DNA (APP (NM_000484) forward: 5′-AGG ACT GAC CAC TCG ACC AG-3′, APP reverse: 5′-CGG GGG TCT AGT TCT GCA T-3′, PSEN1 (NM_000021) forward: 5′-AAT AGA GAA CGG CAG GAG CA-3′, PSEN1 reverse: 5′-GCC ATG AGG GCA CTA ATC AT-3′). PGS was dissolved in saline before oral injection and administered to 6-month-old female WT and 5XFAD mice for 3 weeks at 50 mg/kg daily [30]. Animals were randomly divided into four groups for histological analysis: (1) WT + vehicle group (*n* = 4) treated with saline, (2) WT + PGS group (*n* = 5) treated with PGS, (3) 5XFAD + vehicle group (*n* = 5) treated with saline and (4) 5XFAD + PGS group (*n* = 5) treated with PGS. Animal experiments were conducted according to the Guide for the Care and Use of Laboratory Animals (National Institutes of Health Publication No. 85–23, revised 1985) for maintenance, care and treatment and was approved by the Institutional Animal Care and Use Committee of Konyang University (Project identification code: P-20-15-E-01, date: 27 April 2020).

### 2.8. Preparation of Brain Tissue

To prepare brain tissue for histological analysis, the mice were anesthetized by intraperitoneal injection of Avertin (tribromoethanol; Sigma-Aldrich, St. Louis, MO, USA) at 250 μg/kg. After the behavioral experiment was completed, the mice were anesthetized, and cardiac perfusion was performed in 0.05 M PBS and 0.1 M PBS mixed with 4% paraformaldehyde (PFA). Next, the brain was extracted, immersed in 4% PFA, fixed at 4 °C for 20 h and immersed in 30% sucrose in 0.05 M PBS to prevent freezing. The immersed brain tissue was cut into 30-μm-thick coronal sections using a cryostat at −25 °C (Leica Biosystems, Wetzlar, Germany). The cut tissue was stored in 0.05 M PBS buffer containing 25% ethylene glycol and glycerol at 4 °C until immunohistochemical analysis.

### 2.9. Histological Analysis

For thioflavin S (ThS) staining, four and five coronal brain slices were obtained at the level of the cerebral cortex (+1.18 and +0.50 mm to the bregma) and subiculum (−3.08 and −3.80 mm to the bregma), respectively. The brain slices were placed into wells of an acrylic plate and washed three times with 3 mL of PBS for 5 min each. ThS solution was produced by dissolving 0.5% *w*/*v* ThS in 50% ethyl alcohol. The washed brain sections were incubated with ThS solution for 10 min. Subsequently, tissues were washed three times for 5 min with 50% ethyl alcohol and then three times for 5 min with PBS. They were mounted on slides and cover slipped with Flourished Mounting Medium (Sigma-Aldrich).

For immunohistochemical analysis of oxidative stress, neuroinflammation and neuronal loss, brain slices were placed into wells and rinsed with 3 mL of PBS three times for 5 min. The brain slices were subsequently incubated overnight at 4 °C in PBS containing 0.5 mg/mL BSA, 0.3% Triton X-100, and the following primary antibodies: mouse-anti 4 hydroxynonenal (4 HNE) antibody (1:200; Abcam Cambridge, MA, USA), mouse anti-neuronal nuclei (NeuN) antibody (1:1000; Merck KGaA, San Diego, CA, USA), goat anti-ionized calcium binding adapter molecule 1 (Iba-1) antibody (1:500; Abcam) and rat anti-glia fibrillary acidic protein (GFAP) antibody (1:1000; Thermo Fisher Scientific, Waltham, MA, USA). After washing in PBS three times for 5 min each, the sections were incubated with a secondary antibody for 50 min at room temperature: donkey Alexa 488-conjugated anti-mouse IgG (1:300), donkey Alexa 488-conjugated anti-goat IgG (1:200) and donkey Alexa 594-conjugated anti-rat IgG (1:300) (all from Thermo Fisher Scientific Inc.)

To amplify the 4 HNE immunofluorescence signals, brain tissues were incubated with a biotinylated horse anti-mouse IgG (1:200) secondary antibody for 1 h and incubated with Avidin-Biotin Complex (ABC) solution for 1 h. After washing in PBS three times for 5 min each, the sections were incubated with Alexa Fluor 488–streptavidin (1:300; Thermo Fisher Scientific) for 50 min at room temperature.

To localize the Aβ plaque and astrocytes, microglia, neuronal nuclei and oxidative stress protein, brain tissues were incubated overnight at 4 °C in PBS containing 0.5 mg/mL BSA, 0.3% Triton X-100, and the primary antibodies 4 HNE, NeuN, Iba-1 and GFAP. The next day, the sections were washed in PBS three times for 5 min each and the sections were stained with 0.05% ThS in PBS over 8 min. Subsequently, tissues were washed twice for 1 min each with 50% ethyl alcohol and then once for 3 min with distilled water. Finally, the sections were coverslipped using Flourished Mounting Medium.

### 2.10. Image Acquisition and Analysis

To quantify immunoreactivity, images were obtained with a Zeiss LSM 700 microscope (Carl Zeiss AG, Oberkochen, Germany) and analyzed using ImageJ software (National Institutes of Health, Bethesda, MD, USA). We selected layers 5–6 of the motor cortex to analyze quantitatively the signals of ThS and NeuN. Th S-positive plaques were quantified as area fractions of the motor cortex and NeuN, Iba-1 and GFAP-positive cells were counted as the number of positive cells per mm^2^ of the subiculum. Lastly, 4 HNE immunoreactivity was quantified as the fluorescence intensity in the subiculum.

### 2.11. Statistical Analysis

All analyses were randomly executed in a blinded manner for individual groups. Data are displayed as means ± standard errors of the mean (SEM). *t*-tests and one-way analysis of variance (ANOVA) were conducted using Prism 7.0 software (GraphPad Software, Inc., La Jolla, CA, USA), followed by Tukey’s post hoc analysis for two or more groups. Statistical significance was considered at *p* < 0.05 (* *p* < 0.05, ** *p* < 0.01 and *** *p* < 0.001).

## 3. Results

### 3.1. Analysis of Platycoside E and Platycodin D

**Figure 1:**
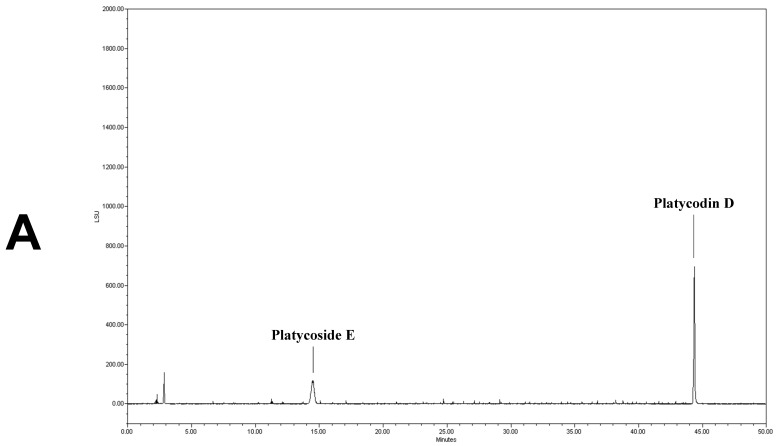

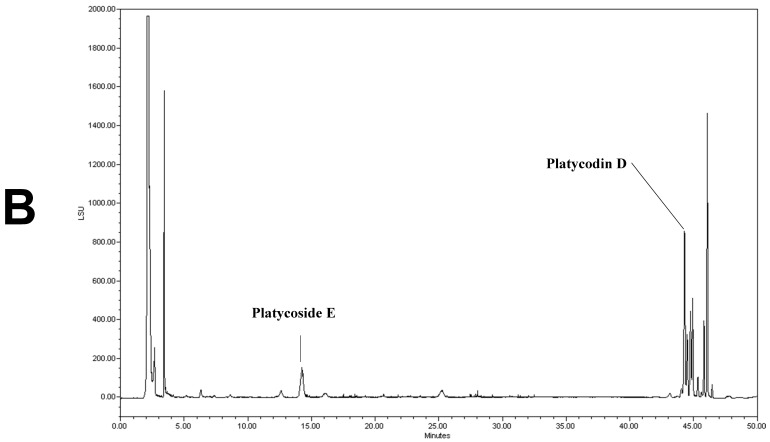

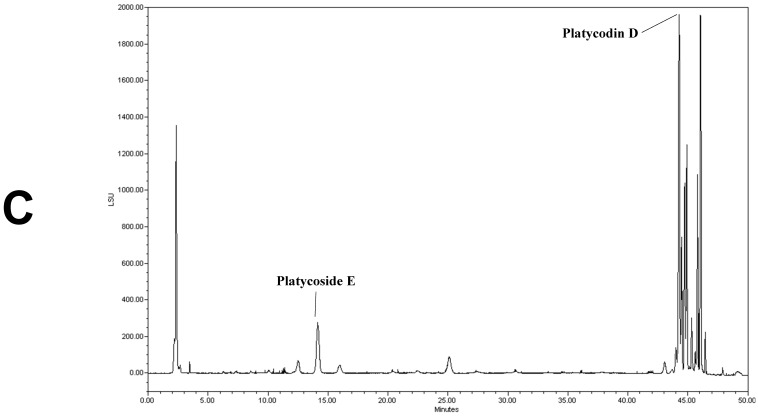
High-performance liquid chromatography with evaporative light scattering detection analysis of *Platycodon grandiflorum* marker saponins. The HPLC chromatograms show the retention times and peaks of platycoside E and platycodin D. (**A**) HPLC profile of a standard solution containing platycoside E and platycodin D (Nam et al. [29]). (**B**) HPLC profile of *Platycodon grandiflorum* extract (PGE). (**C**) HPLC profile of *Platycodon grandiflorum* crude saponin (PGS).

We determined the contents of platycoside E and platycodin D by HPLC with ELSD (Figure 1). Platycoside E and platycodin D were detected at 14.2 and 44.3 min, respectively. The respective platycoside E and platycodin D levels were 5.4 ± 0.1 and 6.5 ± 0.6 mg/g in PGE, and 30.1 ± 1.6 and 41.0 ± 2.9 mg/g in PGS, on a dry extract basis (Figure 1B,C). Therefore, the *P. grandiflorum* root saponins were efficiently concentrated into PGS.

### 3.2. Protective Effect of PGS against Aβ-Induced HT22 Cell Injury

**Figure 2:**
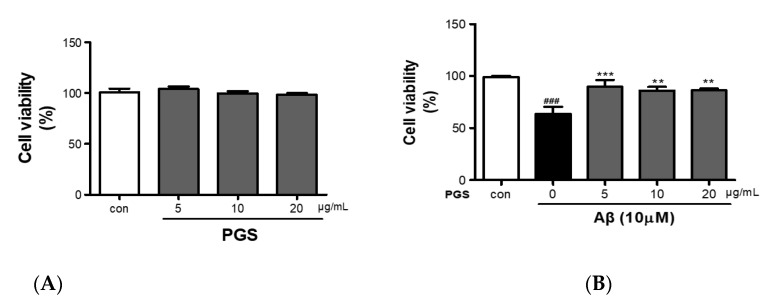
Inhibitory effect of PGE on Aβ-induced oxidative stress in HT22 mouse hippocampal neurons. (**A**) HT22 cells were treated with PGS (5, 10 and 20 μg/mL) and control (0.2% DMSO) for 48 h. (**B**) HT22 cells were treated with PGS (5, 10 and 20 μg/mL) and then with 10 μM Aβ for 48 h. Significance was determined by one-way ANOVA with Tukey’s post hoc multiple comparison test. Data are means ± standard errors of the mean (SEM). ### *p* < 0.001 significance compared with the control (white bar). ** *p* < 0.01, *** *p* < 0.001 significance compared with Aβ-treated cells (black bar).

PGS (5, 10 and 20 μg/mL) did not show cytotoxicity against HT22 cells (Figure 2A). HT22 cells were treated with Aβ (10 µM) and PGS (5, 10 and 20 μg/mL); the Aβ-treated HT22 cells showed about a 40% reduction in viability (Figure 2B). PGS-treated HT22 cells showed an increase in viability, and PGS at 5 μg/mL significantly inhibited the Aβ-mediated reduction in viability (Figure 2B). Therefore, PGS protects hippocampal-derived neurons from Aβ-induced cytotoxicity.

### 3.3. Effect of PGS on Aβ-Induced ROS Production in HT22 Cells

**Figure 3:**
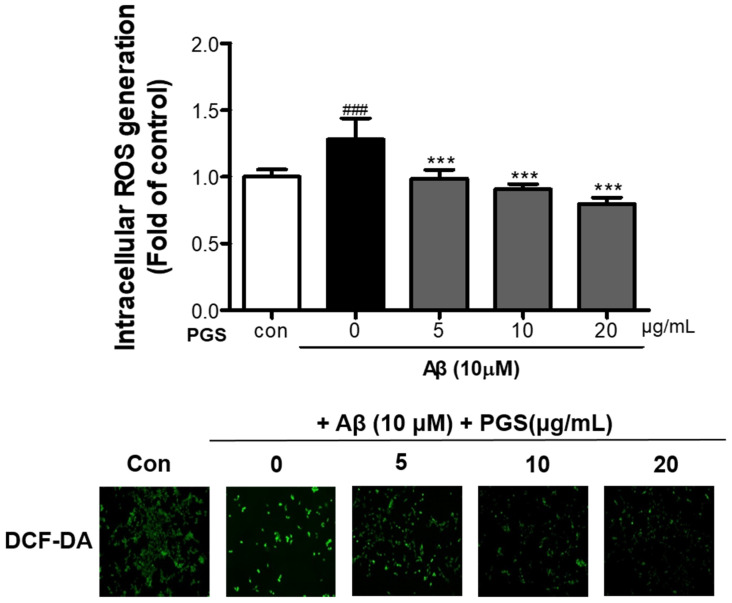
Inhibitory effect of PGS on Aβ-induced oxidative stress in HT22 mouse hippocampal neurons. The cells were treated with PGS (5, 10 and 20 μg/mL) or control (0.2% DMSO) for 24 h and stimulated with Aβ (10 μM) for 4 h. ROS generation in HT22 cells was observed by fluorescence microscopy. Absorbance was measured after DCF-DA staining of cells treated with Aβ or Aβ and PGS. Data are means ± standard errors of the mean (SEM). Significance was determined by one-way ANOVA with Tukey’s post hoc multiple comparison test; ### *p* < 0.001, significance compared with the control (white bar). *** *p* < 0.001, significance compared with Aβ-treated cells (black bar).

To investigate if the cytotoxicity inhibitory effect of PGS is related to amelioration of oxidative stress, we analyzed the effect of PGS on ROS generation by Aβ. Aβ treatment of HT22 neurons increased ROS production compared to the control (Figure 3). However, PGS pretreatment significantly reduced the formation of ROS by Aβ, suggesting that the protective effect of PGS is mediated by a reduction in ROS formation.

### 3.4. Effect of PGS on Antioxidant Enzymes in HT22 Cells

**Figure 4:**
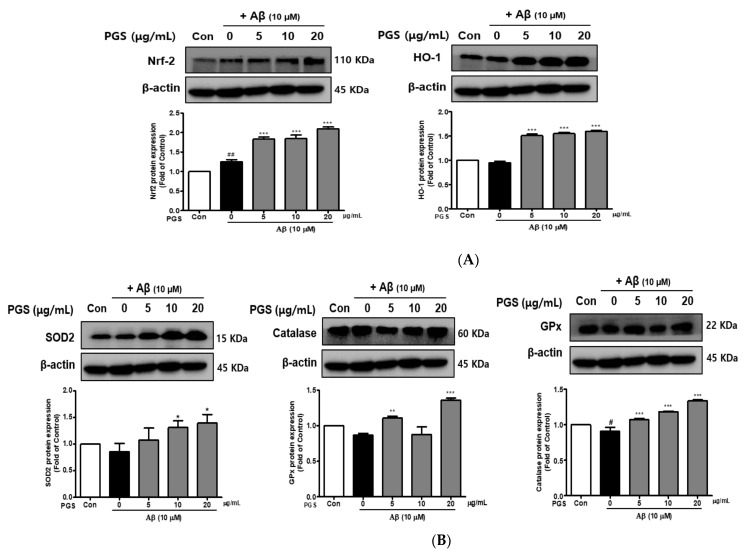
Effect of PGS on the expression of antioxidant enzymes in HT22 cells. Cells were treated with PGS (5, 10 and 20 μg/mL) or control (0.2% DMSO) for 1 h followed by Aβ (10 μM) for 24 h. Proteins were analyzed by western blotting using β-actin as the loading control. (**A**) Nrf2 and HO-1 levels in HT22 cells. (**B**) SOD2, CAT and GPx levels in HT22 cells. Data are means ± standard errors of the mean (SEM). Significance was determined by one-way ANOVA with Tukey’s post hoc multiple comparison test; # *p* < 0.05, ## *p* < 0.01, significance compared with the control (white bar). * *p* < 0.05, ** *p* < 0.01, *** *p* < 0.001, significance compared with Aβ-treated cells (black bar).

The inhibition of ROS generation by PGS was expected to be related to recovery of the antioxidant system, so we investigated the effect of PGS on Nuclear factor E2-related factor 2 (Nrf2)-mediated signaling pathways in HT22 cells. PSG attenuated Aβ-induced neurotoxicity by activating the Nrf2/ARE pathway (Figure 4). The transcription factor Nrf2 was upregulated by PGS (Figure 4A). Additionally, the expression levels of the intracellular antioxidant enzymes HO-1, SOD, CAT, and GPx, target of Nrf2 genes, were upregulated by PGS. Their expression was reduced by Aβ alone, compared to the control, but was recovered by PGS in HT22 cells (Figure 4B). Therefore, the inhibitory effect of PGS on Aβ-induced ROS generation is related to the expression of major antioxidant enzymes in HT22 cells.

### 3.5. Effect of PGS on NF-κB Activation in Aβ-Induced HT22 Cells

**Figure 5:**
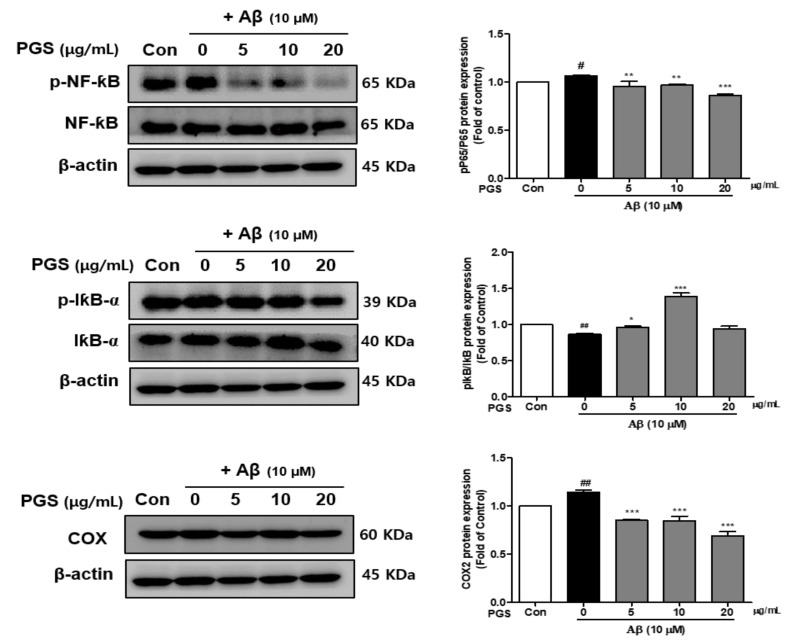
Effect of PGS on NF-κB activation, and the p-NF-κB, NF-κB and I-κBα, p-IκBα and COX protein levels in Aβ-induced HT22 cells. The cells were treated with PGS (5, 10 and 20 μg/mL) or control (0.2% DMSO) for 1 h followed by Aβ (10 μM) for 24 h. Proteins were analyzed by western blotting using β-actin as the loading control. Data are means ± standard errors of the mean (SEM). Significance was determined by one-way ANOVA with Tukey’s post hoc multiple comparison test; # *p* < 0.05, ## *p* < 0.01, significance compared with the control (white bar). * *p* < 0.05, ** *p* < 0.01, *** *p* < 0.001, significance compared with Aβ-treated cells (black bar).

Nuclear factor-kappa-B (NF-κB) is a transcription factor that regulates the expression of several inflammatory cytokines, COX-2 and iNOS genes, and is composed of p50 and p65 subunits [31]. The effects of PGS on the activation of NF-κB and I-κBα, a negative regulator of NF-κB, were investigated. The phosphorylation (p-NF-κB/NF-κB) of NF-κB (p65 subunit) was increased in the Aβ group, which was suppressed by PGS (Figure 5). The phosphorylation of IκBα (p-IκBα) was decreased by Aβ, and was recovered by PGS. Moreover, COX-2, an NF-κB target gene, was upregulated by Aβ, an effect inhibited by PGS. PGS significantly inhibited the activation and intranuclear transport of NF-κB induced by Aβ treatment by phosphorylating IκBα (Figure 5). Therefore, PGS blocks the Aβ-induced activation of the NF-κB inflammatory signaling pathway.

### 3.6. Effect of PGS on Apoptotic Protein Expression in Aβ-Induced HT22 Cells

**Figure 6:**
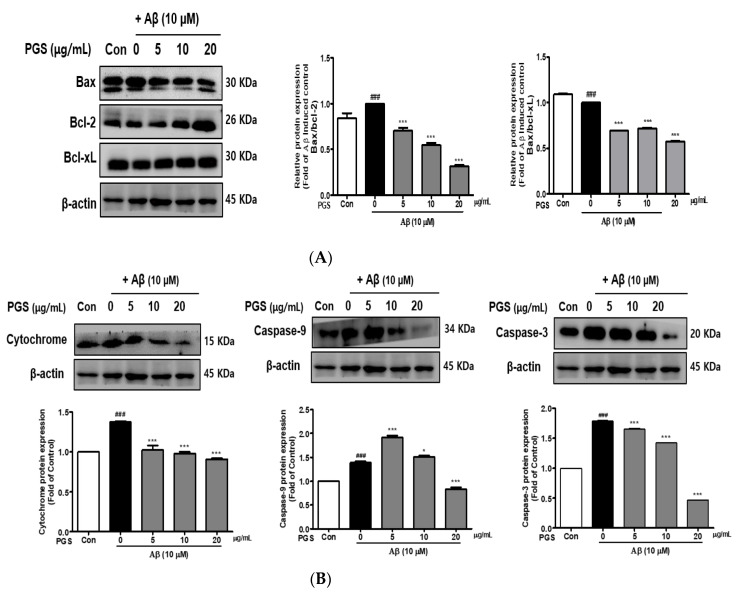
Effect of PGS on Aβ-induced Bcl-2 family protein expression in HT22 cells: PGS protects HT22 cells from Aβ-induced apoptosis. Cells were treated with PGS (5, 10 and 20 μg/mL) or control (0.2% DMSO) for 1 h followed by Aβ (10 μM) for 24 h. Proteins were analyzed by western blotting using β-actin as the loading control. (**A**) Bax/Bcl-xL and Bax/Bcl-2 protein expression in the mitochondria. (**B**) Cytochrome c, caspase-9 and -3 expression in mitochondria. Data are means ± standard errors of the mean (SEM). Significance was determined by one-way ANOVA with Tukey’s post hoc multiple comparison test; ### *p* < 0.001, significance compared with the control (white bar). * *p* < 0.05, *** *p* < 0.001, significance compared with Aβ-treated cells (black bar).

The expression of Bax, a pro-apoptotic protein, was increased by Aβ in HT22 cells. In contrast, the expression of Bcl-2 and Bcl-xL, members of the Bcl-2 family, was decreased. However, the expression of Bax was suppressed and that of Bcl-2 and Bcl-xL was increased by PGS (Figure 6A). Cytochrome c induces apoptosis by triggering the activation of caspase-9 and -3. The expression of cytochrome c, caspase-9 and caspase-3 was upregulated by Aβ, which was significantly inhibited by PGS in a dose-dependent manner (Figure 6B), confirming the protective effect of PGS on Aβ-induced apoptosis. PGS inhibited the release of cytochrome c and the expression of caspase-9 and -3. Therefore, PGS attenuates Aβ-induced apoptosis by regulating the expression of Bcl-2 family proteins, thereby exerting a neuroprotective effect by inhibiting the mitochondrial apoptosis pathway.

### 3.7. Effect of PGS on the MAPK Signaling Pathway in Aβ-Induced HT22 Cells

**Figure 7:**
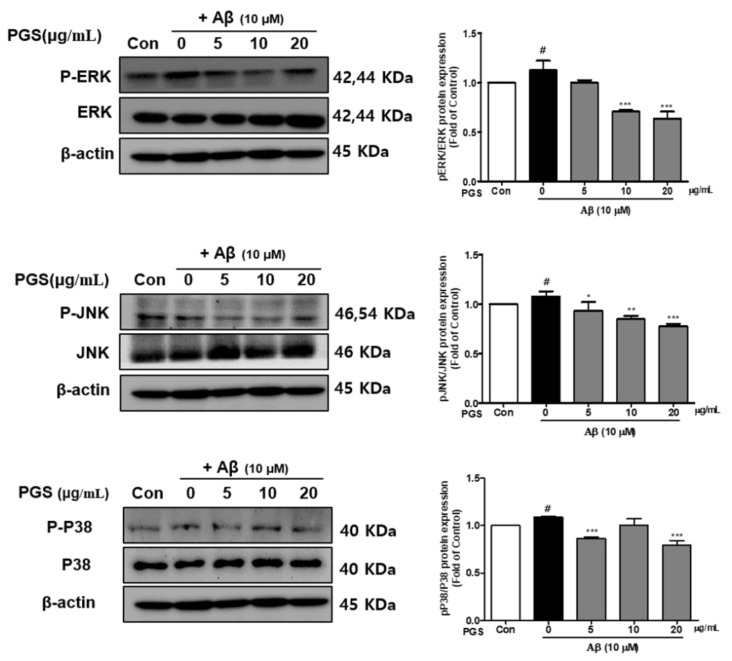
Effect of PGS on MAPK expression in Aβ-induced HT22 cells. Cells were treated with PGS (5, 10 and 20 μg/mL) or control (0.2% DMSO) for 1 h followed by Aβ (10 μM) for 24 h. Proteins were analyzed by western blotting using β-actin as the loading control. Data are means ± standard errors of the mean (SEM). Significance was determined by one-way ANOVA with Tukey’s post hoc multiple comparison test; # *p* < 0.05, significance compared with the control (white bar). * *p* < 0.05, ** *p* < 0.01, *** *p* < 0.001, significance compared with Aβ-treated cells (black bar).

Mitogen-activated protein kinases (MAPKs) include extracellular signal-regulated kinase 1/2 (ERK 1/2), c-Jun N-terminal kinases (JNK) and p38 kinases and are activated by toxins or growth factors, inducing inflammation and apoptosis [32]. Phosphorylation of the MAPKs p38, ERK and JNK was increased by Aβ and inhibited by PGS in HT22 cells. PGS suppressed the activation (p-p38/p38, p-ERK/ERK and p-JNK/JNK) of p38, ERK and JNK (Figure 7). Therefore, PGS downregulates MAPK signaling by inhibiting p38, ERK and JNK activation in Aβ-induced HT22 cells, attenuating inflammation and apoptosis.

### 3.8. PGS Inhibits the Accumulation of Aβ in the Brain of 5XFAD Mice

**Figure 8:**
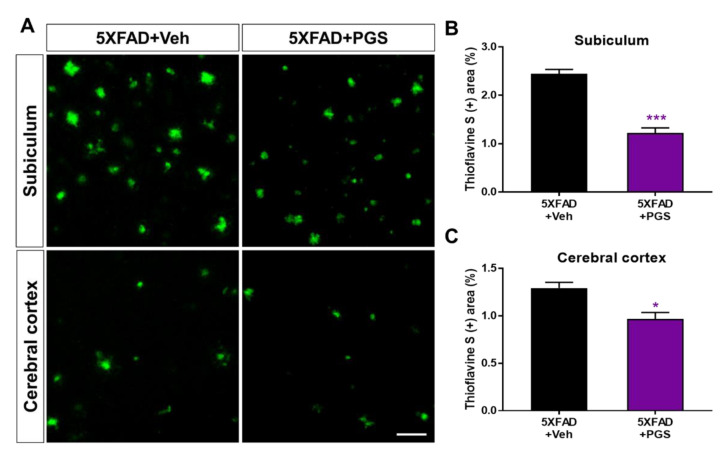
Effect of PGS on amyloid beta (Aβ) plaque deposition in the subiculum and cerebral cortex of Aβ-overexpressing mice. (**A**) Fluorescence signals of thioflavin S (ThS) in the subiculum and cerebral cortex of vehicle- and PGS-treated 5XFAD mice. The PGS-treated 5XFAD mice had a significantly decreased ThS-positive area (%) in the subiculum (**B**) and cerebral cortex (**C**) compared with vehicle-treated 5XFAD mice. The vehicle group was administered saline at the same volume as PGS. Data are means ± standard errors of the mean (SEM). Scale bar = 50 μm. * *p* < 0.05 and *** *p* < 0.001, significant difference compared with vehicle-treated 5XFAD mice (black bar).

Aβ is an essential molecule in AD, inducing neurotoxicity via Aβ aggregates, Aβ oligomers and fibrils [33]. To investigate the effect of PGS on Aβ accumulation, we performed histochemical staining for Aβ in the subiculum and cerebral cortex of 5XFAD mice (Figure 8A). The PGS-treated 5XFAD mice had significantly reduced ThS-positive areas in the subiculum and the cerebral cortex compared with the vehicle-administrated 5XFAD mice (Figure 8B,C). Therefore, PGS ameliorates the accumulation of Aβ in the AD brain.

### 3.9. PGS Alleviates Oxidative Damage in the Brain of Aβ-Overexpressing Transgenic Mice

**Figure 9:**
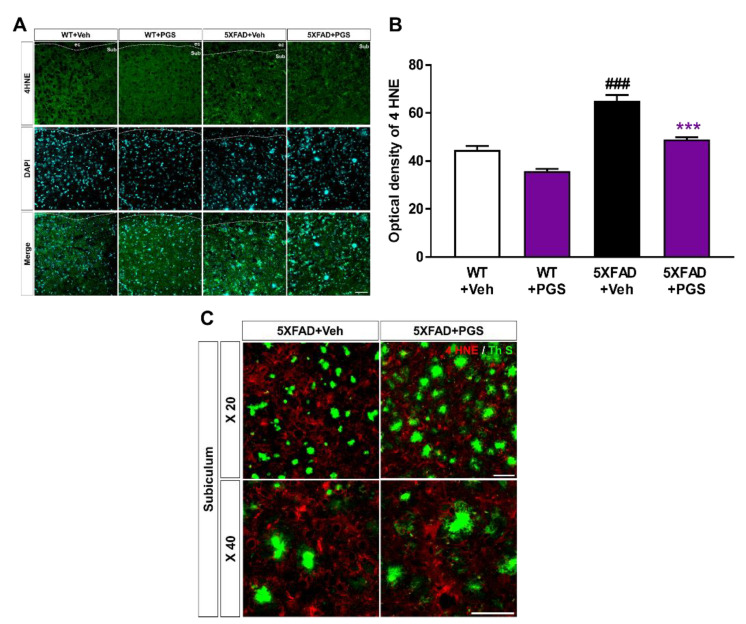
Effect of PGS on oxidative damage in the subiculum of 5XFAD mice. (**A**) Immunoreactivity of 4 hydroxynonenal (4 HNE) in the subiculum of wild-type (WT) and 5XFAD mice treated with vehicle and PGS. (**B**) Vehicle-treated 5XFAD mice showed an increased optical density of 4 HNE in the subiculum compared with vehicle-treated WT mice. However, PGS-treated 5XFAD mice showed a significantly reduced optical density of 4 HNE in the subiculum compared with vehicle-treated 5XFAD mice. (**C**) Extended images of the localization with ThS stained-plaques and oxidative stress protein in the subiculum of the vehicle- and PGS-treated 5XFAD mice. The vehicle group was administered saline in the same volume as PGS. Data are means ± SEM. Scale bar = 50 μm. ### *p* < 0.001, significant difference compared with vehicle-treated WT mice (white bar). *** *p* < 0.001, significant difference compared with vehicle-treated 5XFAD mice (black bar).

The AD brain shows considerable oxidative damage, which is characterized by an imbalance between ROS and antioxidative defenses, associated with the abnormal deposition of Aβ [34,35]. In addition, oxidative damage is responsible for neuronal loss [36]. To investigate the antioxidant effect of PGS in the brain, we evaluated the expression of oxidative damage proteins by staining for anti-4 HNE in the subiculum of 5XFAD mice (Figure 9A). The optical density of subiculum was increased in vehicle-treated 5XFAD mice compared with vehicle-treated WT mice. PGS-treated 5XFAD mice showed a significant decrease in optical density compared to vehicle-treated 5XFAD mice (Figure 9B). Moreover, we revealed that the oxidative stress proteins were localized with Aβ plaques (Figure 9C). Therefore, PGS alleviates oxidative damage in the AD brain.

### 3.10. PGS Decreases Neuroinflammation in the Subiculum of 5XFAD Mice

**Figure 10:**
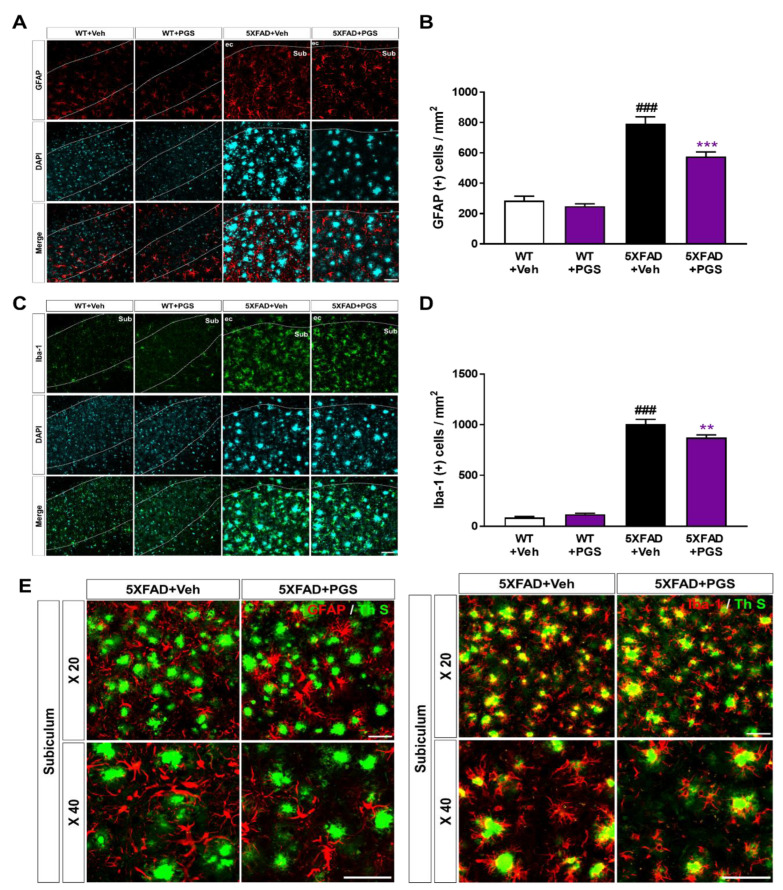
Effect of PGS on neuroinflammation in the subiculum of 5XFAD mice. (**A**) Immunoreactivity of glia fibrillary acidic protein (GFAP) in the subiculum of WT and 5XFAD mice treated with vehicle and PGS. (**B**) The vehicle-treated 5XFAD mice had more GFAP-positive astrocytes in the subiculum than the vehicle-treated WT mice. The PGS-treated 5XFAD mice had significantly fewer GFAP-positive astrocytes in the subiculum compared with vehicle-treated 5XFAD mice. (**C**) Immunoreactivity of ionized calcium binding adapter molecule 1 (Iba-1) in the subiculum of WT and 5XFAD mice treated with vehicle and PGS. (**D**) The vehicle-treated 5XFAD mice had an increased number of Iba-1-positive microglia in the subiculum compared with vehicle-treated WT mice. PGS-treated 5XFAD mice had a significantly decreased number of Iba-1-positive microglia in the subiculum compared with vehicle-treated 5XFAD mice. (**E**) Magnified images of the localization with ThS stained-plaques and astrocytes and microglia in the subiculum of the vehicle- and PGS-treated 5XFAD mice. The vehicle group was administered saline in the same volume as PGS. Data are means ± SEM. Scale bar = 50 μm. ### *p* < 0.001, significant difference compared with vehicle-treated WT mice (white bar). ** *p* < 0.01 and *** *p* < 0.001, significant difference compared with vehicle-treated 5XFAD mice (black bar).

Aβ deposition induces an inflammatory response, such as activation of microglia and astrocytes, contributing to AD development and progression [37]. To examine the effect of PGS on the glial response in the AD brain, we conducted immunofluorescence staining using antibodies against glial fibrillary acidic protein (GFAP) for astrocytes and ionized calcium-binding adapter molecule 1 (Iba-1) for microglia in the subiculum of 5XFAD mice (Figure 10A,C). The number of GFAP-positive neurons per mm^2^ in the subiculum of vehicle-treated 5XFAD mice was increased compared with that in vehicle-treated WT mice. Surprisingly, PGS-treated 5XFAD mice had a significantly decreased number of GFAP-positive cells than vehicle-treated 5XFAD mice (Figure 10B). Moreover, the number of Iba-1-positive neurons per mm^2^ in the subiculum of vehicle-treated 5XFAD mice was increased compared with that in vehicle-treated WT mice. Surprisingly, PGS-treated 5XFAD mice had a significantly lower number of Iba-1-positive cells than vehicle-treated 5XFAD mice (Figure 10D). Moreover, we found that astrocytes and microglia were localized around Aβ plaques (Figure 10E). Particularly, Iba-1-positive microglia colocalized with Aβ plaques. Taken together, these findings suggest that PGS significantly alleviated astrogliosis and microgliosis in the AD brain.

### 3.11. PGS Ameliorates Neurodegeneration in the Cerebral Cortex of an AD Animal Model

**Figure 11:**
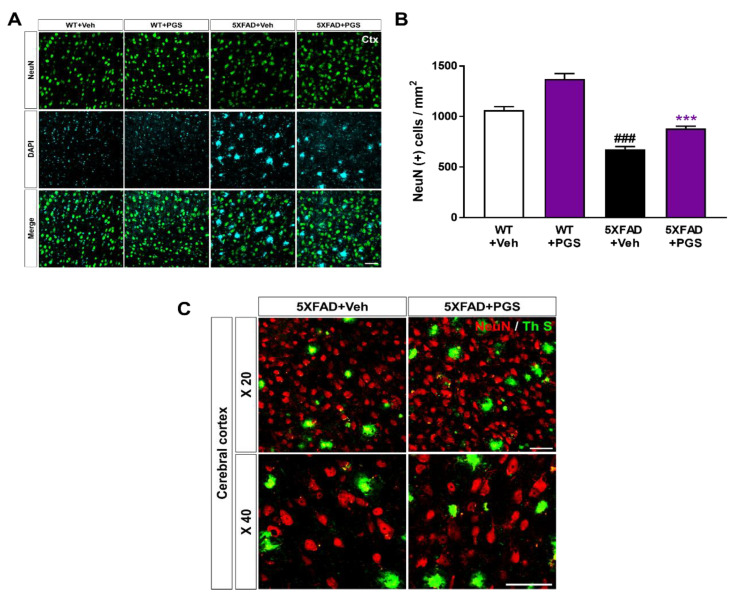
Effect of PGS on neurodegeneration in the cerebral cortex of an animal model of AD. (**A**) Immunoreactivity of neuronal nuclei (NeuN) in the cerebral cortex of WT and 5XFAD mice treated with vehicle and PGS. (**B**) Vehicle-treated 5XFAD mice had a reduced number of NeuN-positive cells in the cerebral cortex compared with vehicle-treated WT mice. Interestingly, PGS-treated 5XFAD mice had a significantly increased number of NeuN-positive cells in the cerebral cortex compared with vehicle-treated 5XFAD mice. (**C**) Magnified images of the localization with ThS stained-plaques and neuronal nuclei in the cerebral cortex of the vehicle- and PGS-treated 5XFAD mice. The vehicle group was administered saline in the same volume as PGS. Data are means ± SEM. Scale bar = 50 μm. ### *p* < 0.001, significant difference compared with vehicle-treated WT mice (white bar). *** *p* < 0.001, significant difference compared with vehicle-treated 5XFAD mice (black bar).

Neuronal loss in AD is associated with impairment of cognitive function [38]. To investigate whether PGS has a neuroprotective effect, we performed immunohistochemical staining of the subiculum in WT and 5XFAD mice using a NeuN antibody (Figure 11A). The number of NeuN-positive neurons per mm^2^ in the deep cortical layers of vehicle-treated 5XFAD mice was reduced compared with that in vehicle-treated WT mice. Surprisingly, PGS-treated 5XFAD mice had a significantly higher number of NeuN-positive cells than vehicle-treated 5XFAD mice (Figure 11B). We showed plaques localized with the neuronal population in the cerebral cortex of the vehicle- and PGS-treated 5XFAD mice (Figure 11C). Therefore, PGS can rescue neuronal loss in brains with Aβ.

## 4. Discussion

Oxidative stress, inflammation and apoptosis are involved in the progression of AD and other neurodegenerative diseases [39]. Among the pathological features of AD, extracellular amyloid plaques are found in areas related to cognitive functions in the brain, such as the hippocampus and cerebral cortex. Aβ is essential for activating oxidative mediators. Excess accumulation of Aβ not only induces neuronal cell death by activating ROS generation and oxidative neurotoxicity but is also closely related to acute and chronic neurodegenerative diseases [40,41]. The inflammatory response induced by Aβ deposition activates microglia and astrocytes, which release inflammatory cytokines, such as IL-β, TNF-α and COX-2. In a recent study, peroxisome proliferator-activated receptor (PPARγ), a ligand-dependent nuclear hormone receptor transcription factor, exerted anti-inflammatory effects by inhibiting the activation of microglia and astrocytes and reducing the production of pro-inflammatory cytokines. The inhibitory effect of PPAR-γ on pro-inflammatory genes antagonizes NF-κB action, which is an essential regulator of the upregulation of expression of many pro-inflammatory cytokines and inducible effector enzymes involved in inflammatory processes [42,43]. Methods to eliminate Aβ or ameliorate neuroinflammatory responses are promising pathways for developing drugs for neurodegenerative diseases. Aβ_25–35_, used in the experiment, is a short fragment derived from amyloid precursor protein and has neurotoxic effects similar to those of Aβ_1–40/1–42_. Therefore, it is considered suitable for Aβ-induced cytotoxicity in the hippocampus, cerebral cortex and mouse hippocampus-derived HT22 cells. HT22 cells are a sublineage derived from the original immortalized parental HT4 cells in primary mouse hippocampal neuronal cell cultures and serve as a good model for studying AD pathology in vitro [44]. We investigated the effect of PGS on Aβ-induced neuronal injury in vitro and in vivo in HT22 cells and 5XFAD mouse brains.

PG root has multiple active ingredients, such as phenolic acid, flavonoids and sterols. These include 17 saponins [45,46], among which platycodin D, deapi-platycodin D, platycoside E, polygalacin D2 and polygalacin D are important. Platycoside E ameliorated ethanol-induced cognitive impairment in mice [47]. Platycodin A had a neuroprotective effect by inhibiting glutamate-induced toxicity and an anti-inflammatory effect by suppressing cytokine and chemokine secretion and NF-κB activation [48]. The triterpene saponin platycodin D has an anti-inflammatory effect [49] and polygalacin D promotes neurite outgrowth of neuronal cells and protects neuronal cells against oxygen-glucose deprivation and ischemia [50,51]. Fu et al. investigated the anti-inflammatory effect of polygalacin D in LPS-induced primary rat microglia and showed that it significantly inhibited ROS, TNF-α, IL-6 and IL-1β production [52].

A limitation of this research is that we did not show the bioactivities of each PGS saponin. However, our study showed that *P. grandiflorum* root saponins, which are major bioactive molecules of *P. grandiflorum*, were efficiently concentrated in PGS. In previous studies, *P. grandiflorum* root saponins, including platycodin D and platycoside E, had protective effects against neurotoxicity, suggesting that PGS components can protect neurons against Aβ-induced neurotoxicity effectively.

**Figure 12:**
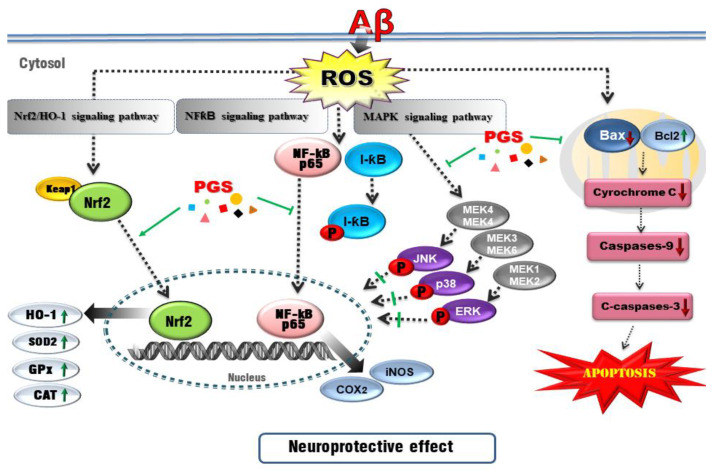
Schematic of the mechanisms of the amelioration by PGS of Aβ-induced neuronal cell death. Red, Aβ-induced pathologies; green lines/arrows, amelioration by PGS in neurons.

The nuclear factor erythroid-2-related factor 2 (Nrf2) pathway, a critical signaling pathway for ROS detoxification in the brain, constitutes an important cellular defense mechanism against oxidative stress injury [53]. Nrf2 regulates the cellular antioxidant system [54]. When cells are exposed to neurodegenerative disease-mediated oxidative stress, phosphorylation of Keap1 causes Nrf2 to migrate to the nucleus, where it binds to the small Maf protein and the antioxidant responsive element (ARE). Antioxidant enzymes such as SOD-1, SOD-2, Catalase, GPx or heme oxygenase-1 (HO-1) respond to ROS by activating the Nrf2 signaling pathway in HT22 cells [55,56]. In this study, PGS enhanced activation of the Nrf2/ARE pathway and increased the expression of HO-1, SOD, CAT and GPx (Nrf2 target genes), thereby significantly reducing Aβ-induced ROS production in HT22 cells. This suggests that PSG attenuates Aβ-induced oxidative stress by activating the Nrf2/ARE pathway (Figure 12).

Oxidative stress can trigger apoptotic pathways (e.g., Bax, Bad and caspases) and Aβ peptide and tau phosphorylation toward neuronal cell death. Since brain neurons consume much energy and are highly dependent on ATP, mitochondrial dysfunction induces apoptosis [57]. Aβ-induced oxidative stress in neurons damages the inner mitochondrial membrane, and Bax (which modulates mitochondrial membrane permeability) increases the secretion of cytochrome c. Cytochrome c release into the cytoplasm activates the cysteine proteases caspase-9 and -3, resulting in neuronal cell death [58,59]. Aβ treatment induced apoptosis of HT22 cells by promoting accumulation of ROS. However, ROS generation and apoptosis were reduced by PGS. Aβ increased the expression of Bax, an effect reversed by PGS. Additionally, PGS reduced Bax/Bcl-2 and Bax/Bcl-xL, leading to downregulation of cytochrome c, caspase-9 and caspase-3. PGS reduced the expression of Bax, suppressing the loss of mitochondrial membrane potential and preventing cytochrome c release. As a result, the activation of the sub-signal caspase group was inhibited, preventing induction of apoptosis (Figure 6 and Figure 12).

Recently, AD research has focused on inflammation. The increase in oxidative stress caused by aging and damage induces transcription factors such as NF-κB, activating the production of cytokines related to the inflammatory response, AD, dementia, arteriosclerosis, kidney disease and cardiovascular disease [60,61]. NF-κB is an early response factor that activates proinflammatory factors, COX-2 and iNOS genes, and is composed of subunits p50 and p65 [62]. The COX-2 gene promoter has two binding sites for NF-κB, which positively regulate its expression during inflammation [63]. COX-2 inhibitors function by inactivating NF-κB [64]. I-κB exists in an inactive state under normal conditions, inhibiting the activation of NF-κB. However, an external inflammatory stimulus triggers I-κB phosphorylation and degradation by ubiquitination, by IKKα/β. Subsequently, the NF-κB (p50/p65) dimer is activated and migrates from the cytoplasm to the nucleus. Zhang et al. reported that *P. grandiflorum* saponins showed an anti-inflammatory effect by inhibiting NF-κB activation in mice and alleviating the cisplatin-induced increase in iNOS and COX-2 [65]. We confirmed that activation of NF-kB by Aβ was significantly inhibited by PGS, and I-κBα expression was increased (Figure 5). PGS inhibited NF-κB signaling by modulating the activation of NF-κB and IκBα by Aβ. Therefore, PGS can attenuate Aβ-induced neuroinflammation by downregulating NF-κB signaling (Figure 12).

The intracellular inflammatory response to Aβ is related to the activation of NF-κB by Toll-like receptor 4 (TLR4) receptor on the cell membrane. Interleukin-1 receptor-associated kinase 1/4 (IRAK1/4), as well as MAPK pathway proteins, such as p38, JNK and ERK 1/2, are associated with NF-κB activation [66]. MAPKs are involved in normal cell division, proliferation and differentiation regulation and are controlled by phosphorylation. In addition, MAPKs induce apoptosis [67,68]. In this study, Aβ-induced activation of p38, JNK and ERK 1/2 was suppressed by PGS. Therefore, PGS exerts anti-inflammatory and anti-apoptotic effects by inhibiting MAPK signaling in Aβ-treated HT22 cells (Figure 12).

Neuronal loss in AD is closely related to cognitive impairment [69], and Aβ deposition induces inflammatory responses (such as microglia and astrocyte activation), contributing to AD development and progression [70]. We demonstrated that PGS ameliorated Aβ-mediated pathologies in animal models of AD. PGS significantly ameliorated Aβ accumulation (Figure 8) and mitigated oxidative damage in the AD brain (Figure 9). In addition, PGS-treated 5XFAD mice had significantly fewer GFAP-and Iba-1-positive cells and more NeuN-positive cells than vehicle-treated 5XFAD mice (Figure 10 and Figure 11). This suggests that PGS can attenuate Aβ-induced neuroinflammation and neuronal loss in AD brain.

In this study, PGS attenuated Aβ-induced damage in hippocampal HT22 cells and 5XFAD mice, an animal model of AD. Therefore, PGS has potential for preventing degenerative neurological diseases such as AD.

## 5. Conclusions

PGS prevented Aβ-induced neurotoxicity in hippocampal cells and ameliorated the Aβ-mediated pathologies in animal models of AD. Our in vitro results showed that PGS reduced Aβ-induced oxidative stress and neuronal apoptosis via antioxidant, anti-inflammatory and anti-apoptotic signaling pathways. In an AD animal model, PGS exerted antioxidant, anti-inflammatory and anti-apoptotic effects on Aβ-induced nerve damage in vivo. Taken together, our findings suggest that PGS has potential for preventing degenerative brain neurological diseases such as AD.

## Data Availability

Data is contained within the article.

## Abbreviations

Aβ	amyloid beta
AD	Alzheimer’s disease
CAT	catalase
COX-2	cyclooxygenase 2
GPx	glutathione peroxidase
HO-1	heme oxygenase-1
HT22	hippocampal neuronal cells
IKBα	NF-κB inhibitor
Keap1	Kelch-like ECH related protein 1
MAPK	mitogen-activated protein kinase
NF- κB	nuclear factor kappa B
Nrf2	nuclear factor erythroid 2 related factor 2
ROS	reactive oxygen species
SOD	superoxide dismutase
